# Leg length measures appear inaccurate in the early phase following total hip arthroplasty

**DOI:** 10.1038/s41598-021-02684-3

**Published:** 2021-12-01

**Authors:** Maria Anna Smolle, Stefan Franz Fischerauer, Michael Maier, Patrick Reinbacher, Jörg Friesenbichler, Paul Ruckenstuhl, Maria Grandesso, Andreas Leithner, Werner Maurer-Ertl

**Affiliations:** 1 Department of Orthopaedics and Trauma, Medical University of Graz, Auenbruggerplatz 5, 8036 Graz, Austria; 2 Dipartimento Universitario Clinico Di Scienze Mediche, Chirurgiche E Della Salute, Universitá Degli Studi Di Trieste, Strada Di Fiume, 447, Trieste, Italy

**Keywords:** Medical research, Outcomes research

## Abstract

The aims of this study were to (1) assess reliability of leg length discrepancy (LLD) measurements at different anatomical landmarks, (2) longitudinally investigate LLD in patients within the first year following total hip arthroplasty (THA) and to (3) correlate changes in LLD with functional outcome. Ninety-nine patients with short stem THA (53.3% males, mean age: 61.0 ± 8.1 years) were prospectively included. Upright pelvic anteroposterior (a.p.) radiographs taken at 6 timepoints (preoperatively, discharge, 6, 12, 24, 52 weeks postoperatively) were used to assess LLD at 5 anatomical landmarks (iliac crest, upper sacroiliac joint, lower sacroiliac joint, tear drop figure, greater trochanter). WOMAC and Harris Hip Score (HHS) were obtained preoperatively and at 6 and 52 weeks. LLD measures significantly increased in the initial phase following THA, from discharge to 6 weeks postoperatively and remained constant thereafter. Documentation of LLDs is dependent on measurement site: LLDs varied significantly between trochanter and iliac crest to tear drop figure (*p* < 0.001). Functional assessments did not correlate with the occurrence of LLDs [WOMAC (*p* = 0.252); HHS (*p* = 0.798)]. Radiographic assessment of LLD following THA may not be performed early postoperatively, as measurements appear to inaccurately reflect actual LLDs at this time, potentially due incomplete leg extension and/or inhibited weight-bearing.

## Introduction

Leg length discrepancies (LLDs) are found in at least 60% of healthy individuals, being asymptomatic in most cases^1^. Following total hip arthroplasty (THA), however, changes in leg length over 1.5 cm may cause altered gait pattern, lower back pain, and patient dissatisfaction^2,3^.

Different methods to measure LLD have been established, including the clinical methods absolute and relative LLD measurement, as well as the radiology-based methods trochanteric and standardised-trochanteric LLD measurement^4–6^. However, all these methods are prone to measurement errors, thus impairing inter- and intra-observer-reliability. Although radiologically-based LLD measurements are deemed more accurate considering that specific anatomical landmarks can be determined, varying patient positioning and hip rotation as well as inclination may significantly reduce their reliability^7,8^. Moreover, both trochanteric and standardised-trochanteric measurement only take into account LLD deriving from the leg, rather than also incorporating potential differences in hip center of rotation and pelvic alignment.

Following THA, patients may feel gait differences due to changes in hip anatomy as altered femoral offset with consecutive changes in muscular lever arm and thus relative weakness. Despite preoperative templating and intraoperative comparison of leg lengths, patients may subjectively feel significant LLDs postoperatively. Although LLDs should be addressed early postoperatively by orthopaedic arch support and physiotherapy, proper timing of reliable and reproducible LLD-assessment is warranted.

The aims of the present study were to assess (1) whether LLD measures at typical anatomical landmarks are equally reliable, (2) whether LLD measures on upright pelvic anteroposterior radiographs are changing over time, and (3) whether detected LLDs correlate with functional outcomes.

## Material and methods

One-hundred patients undergoing primary THA with a standardised pressfit cementless short stem hip system (ANA NOVA Proxy® Stem and Alpha® Cup, ImplanTec GmbH, Mödling, Austria) and ceramic bearings were consecutively enrolled to this observational study. All surgeries as corresponding preoperative digital templatings were performed by a single experienced surgeon at our institution in the period between February, 2016 and March, 2017. Ninety-nine patients were eligible for final analysis, including 3 patients with concurrent bilateral THA (6.1%), 2 patients with metachronous bilateral THA (4.0%) and 89 patients with unilateral THA (89.9%). One patient was excluded as statistical outlier with respect to pre-existing leg length difference of more than 5.0 cm due to functional soft tissue contracture that could be corrected during surgery.

Operations were performed through a minimally invasive anterolateral approach in a supine position after preoperative digital templating with mediCAD 2D (*Hectec GmbH, Altdorf bei Landshut, Germany; Version 5.5 (since then updated to Version 6.0, see: *https://www.medicad.eu/en/medicad/medicad-classic). A preoperative single dose antibiotic prophylaxis was given. Patients were allowed to start full weight-bearing on day one after operation. Crutches were prescribed for 6 postoperative weeks.

The study was reviewed and approved by our local institutional review board (EK-Nr. 28-152 ex 15/16). All patients provided informed consent prior to their participation.

### Leg length discrepancy measures

**Figure 1:**
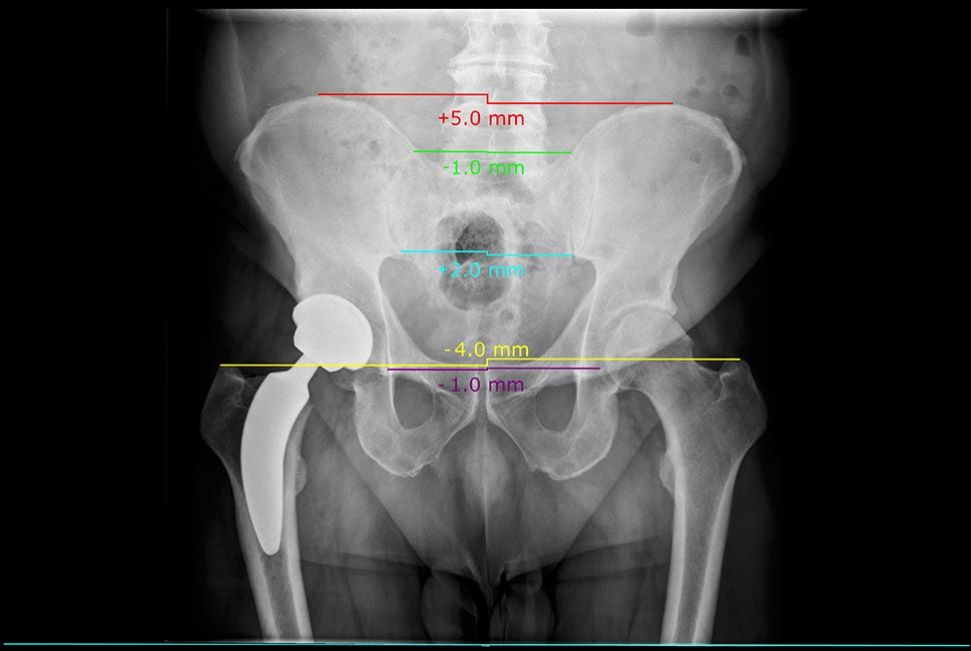
Anatomical landmarks for LLD measures (yellow = trochanter; purple = tear drop figure; blue = lower sacroiliac joint; green = upper sacroiliac joint; red = iliac crest) as measured with mediCAD 2D (*Hectec GmbH)* software.

Preoperatively, LLD was measured using anteroposterior pelvic radiograph in an upright standing position (neutral abduction and flexion, 15° internal rotation; X-ray tube-to-film distance of 150 cm in perpendicular orientation). Likewise, postoperative anteroposterior pelvis radiographs were obtained in the standardised standing position at the time of discharge (after 4–7 days), and after 6, 12, 24, and 52 weeks. A true leg/limb length discrepancy (LLD) between the (to-be) operated and contralateral leg was calculated as the absolute difference from a horizontal line to five different anatomical landmarks: the acetabular tear drop figure, the most inferior portion of the sacroiliac joint, the most superior portion of the sacroiliac joint, the most superior portion of the iliac crest, and the most cranial location of the greater trochanter (Fig. 1). Digital image viewing and measurements were performed on mediCAD 2D (*Hectec GmbH*) by two independent investigators after calibrating the images to either a standardised, 25 mm-diameter calibration ball, or the femoral head of the operated hip, being 32 to 36 mm. Neither of the independent investigators was the operating surgeon.

### Assessment and evaluation of the clinical outcome

Clinical assessment of hip related pain and functional abilities were assessed through the clinician-based Harris Hip Score (HHS) preoperatively, as well as 6 weeks and 12 months postoperatively. The HHS is a frequently used clinician based valid and reliable^9–12^ instrument for the assessment of hip related symptoms and functionality after THA^13–16^. The HHS ranges from 0 to 100 with higher scores reflecting higher function and better outcomes.

The Western Ontario and McMaster Universities Osteoarthritis Index (WOMAC) was used to assess patient-reported pain, stiffness, and physical function. The questionnaire is a widely used, reliable, and validated instrument with high sensitivity to changes in the health status of patients with hip and/or knee related osteoarthritis or joint replacement^17–19^. It comprises 24-items that cover 3 dimensions: pain (5 questions), stiffness (2 questions), and function (17 questions). Patients can choose their answer on an ordinal 11-point Likert-Scale. Higher scores indicate worse pain, stiffness, and functional impairment. We administered a validated German version of the WOMAC^20^.

Orthopaedic arch supports used by patients for actual or perceived LLDs following surgery were documented.

All methods were carried out in accordance with relevant guidelines and regulations. Ethical approval has been obtained by the institutional review board.

### Statistical methods

Statistical analyses were performed using Stata/MP 13.0 (StataCorp, College Station, Texas). Parametric tests were chosen over non-parametric tests as previous simulation studies showed that populations around 100 are "sufficiently large" for the application of parametric tests despite any assumption of normal distribution^21^. We present patients’ characteristics by measures of central tendencies (e.g. proportion, mean [and standard deviation], median [and interquartile ranges, IQR]) as appropriate. Chi-squared tests were performed to assess differences in groups for binary and categorical variables. (Paired) t-tests or Wilcoxon-rank-sum tests were used in case of normally and non-normally distributed continuous variables, respectively. A *p* value < 0.05 was considered as being statistically significant.

Intraclass correlation coefficient (ICC) of the absolute agreement on a two-way random model was used to assess the interrater reliability of LLD measurements at various anatomical landmarks. The interpretation of an average consistency between the two observers is performed as follows: values less than 0.5 indicate poor, values between 0.5 and 0.75 moderate, values between 0.75 and 0.9 good, and values greater than 0.90 excellent reliability^22^.

A multilevel linear mixed effects model for repeated measurements with potentially correlated random LLD intercepts and slopes, nested for anatomical landmarks and using restricted maximum likelihood (REML), was constructed to study the variation in LLD measurements over time (with all LLDs turned into positive values). As in particular LLD measurements taken at the tear drop figure are recommended as a reference point on a.p. pelvic radiographs, they were used as reference to the other anatomical landmark-based measurements within the statistical models^23^. Scientifically relevant demographic variables (age, gender, and BMI) were added to the model to determine potential effects on the outcome. Quadratic terms were added to reflect if effects can rather be described on a linear or quadratic relationship. Coefficients (b), corresponding 95% confidence intervals, standard errors (SEs), z-values and *p* values were provided. Furthermore, multilevel linear mixed effects models for repeated measurements with REML were used to investigate longitudinal changes of clinical outcome assessment (i.e. HHS & WOMAC). Relevant demographic data were added to increase the models’ predictability. LLD measures (from the iliac crest) were transformed to positive values and added to the model to assess a potential independent effect on the functional outcomes. An interaction term between LLD and time accomplished was added to the model to investigate whether a time dependent effect of LLD exists.

There are currently no accepted general standards for sample size calculation in linear mixed effect models. However, it is accepted to recast random effects to simple plot-models and use multivariate linear models with random effects modelled instead as multiple response values for calculations^24^. In such analogy, a sample of 85 participants would enable a regression model with 80% statistical power at an alpha-level set at 0.05 and four predictors to account for 15% or more of the variability in the outcome.

### Ethics approval and consent to participate

The present study has been approved by the Institutional Review Board of the Medical University of Graz (IRB-No. 28-152 ex 15/16). All patients gave their written informed consent prior to being included in this study.

### Consent for publication

As no patient-identifying information is made public, no specific consent with regards to this publication was obtained from patients.

## Results

### General

Table 1 shows the demographic distribution of the study population (N = 99; mean age: 61 years; 46% female, 54% male; mean BMI: 29). Indications for surgery included osteoarthritis in 95 cases (96%) and avascular necrosis of the femoral head in 4 (4%). All patients showed substantive clinical impairments in the preoperative assessments, demonstrating median HHS of 50 (IQR: 42–60). Preoperative LLD measurements were normally distributed at all measurement points and ranged at various hip and pelvis landmarks from − 16.5 mm to 22 mm (Table 1). Postoperatively, 18 patients used orthopaedic arch support for perceived LLD (18.2%), whilst 76 did not (76.8%), and information on use of corrections was missing in 5 patients (5.1%).

**Table 1:** Patient demographics (n = 99).

**Age** (years; mean ± SD)	61.0 ± 8.1
**Sex** (n; %)	
*Female*	46 (46.5%)
*Male*	53 (53.5%)
**Race** (n; %)	
*White*	99 (100%)
**Work status** (n; %)	
*Employed*	36 (36.4%)
*Unemployed*	6 (6.1%)
*Retired*	57 (57.6%)
**BMI** (median, IQR)	28.7 [25.3–31.3]
**Indication for surgery** (n; %)	
*Osteoarthritis*	95 (96.0%)
*Avascular necrosis*	4 (4.0%)
**Preoperative clinical assessment** (median, IQR)	
*Harris Hip Score*	50 [42–60]
*WOMAC Score*	47.1 [36.7–64.6]
**Preoperative LLD** (mm ± SD; (range))*	
*Tear drop figure*	− 0.72 ± 4.2 (− 11 to 10.5)
*Lower Sacroiliac Joint*	− 0.51 ± 4.8 (− 16.5 to 16.5)
*Upper Sacroiliac Joint*	− 0.35 ± 5.1 (− 14.0 to 13.5)
*Iliac Crest*	− 0.68 ± 6.2 (− 11.5 to 17.0)
*Trochanter*	0.88 ± 7.1 (− 15.0 to 22.0)
**Orthopaedic Arch Support for perceived LLD (postop.)** (n; %)	
*No*	76 (76.8%)
*Yes*	18 (18.2%)
*Missing*	5 (5.1%)

*BMI* body mass index, *LLD* leg length difference, *IQR* interquartile range, *WOMAC* Western Ontario and McMaster Universities Osteoarthritis Index.

*Mean of the two observer’s measurements.

### Interobserver variability

Interobserver agreement of LLD measures was strong for all anatomical landmarks. Excellent agreement was achieved for measures at the trochanter (95% CI 0.95–0.96), the iliac crest (95% CI 0.93–0.95), and the upper sacroiliac joint (95% CI 0.90–0.93) [note that observation numbers of iliac crests and upper sacroiliac joints was slightly decreased due to cropped beam projections]. Measures at the lower sacroiliac joint (95% CI 0.88–0.92) and the tear drop Fig. (95% CI 0.89–0.92) revealed good to excellent interobserver agreement.

### Change in LLD over time

**Figure 2:**
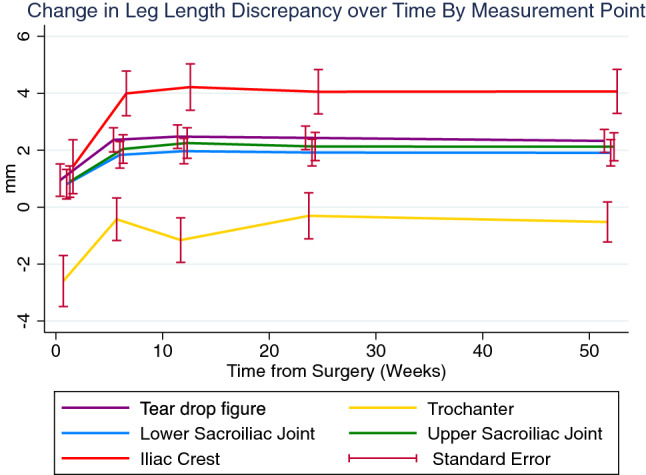
Leg length differences measured at five anatomical landmarks at 1, 6, 12, 24 and 52 weeks after THA. Measures at the tear drop figure and the upper and lower sacroiliac joints were similar, whereas LLD measures at the iliac crest revealed significantly larger values. Interestingly, an LLD increase occurred from week 1 to week 6 at all measure points. This can be referred to relieving postures with unstraightened operated legs at week 1. As reference, measures at the trochanter feature the same progression.

Repeated LLD measures at week 1, 6, 12, 24 and 52 after total hip replacement were performed at five anatomical landmarks, resulting in 2349 measurement values available (126 measurements missing [5.1%]; Table 2). Mean LLD measures and standard errors of the study population over time are demonstrated in Fig. 2. Leg length difference measures at the tear drop figure and the upper and lower sacroiliac joints were nearly congruent, whereas LLD measures at the iliac crest were distinct larger over the entire observation period. Interestingly, an increase of LLD was observed in the entire sample in the initial postoperative phase between week 1 and week 6 and reached a steady plateau subsequently. As the LLD increase between week 1 and 6 is also seen coincidently with measures at the trochanter (note that measures at the trochanter in upright standing positions can be interpreted as reference mark, because THAs do not impact limb lengths distal to the hip joint), inferences may be drawn that patients have not been standing upright with both legs fully straightened, thus eventually leading to hip flexion or pelvic torsion. With measures at week 6, trochanter reference measures reached balanced LLD, indicating that standard upright standing and balanced weight bearing was regained.

**Table 2:** Postoperative LLD measurements (in mm) at different time points as assessed on distinct anatomical landmarks (based on 2349 measurements (depicting the mean of the two observer’s measurements).

LLD (mm)	Week 1	Week 6	Week 12	Week 24	Week 52
*Tear Drop Figure*	0.9 ± 5.5	2.4 ± 4.1	2.5 ± 4.0	2.4 ± 4.0	2.3 ± 4.0
*Trochanter*	− 2.6 ± 8.7	− 0.4 ± 7.1	− 1.2 ± 7.5	− 0.3 ± 7.7	− 0.5 ± 6.9
*Lower Sacroiliac Joint*	0.8 ± 5.0	1.8 ± 4.5	2.0 ± 4.3	1.9 ± 4.5	1.9 ± 4.6
*Upper Sacroiliac Joint*	0.9 ± 5.4	2.0 ± 4.8	2.3 ± 5.2	2.1 ± 4.7	2.1 ± 4.9
*Iliac Crest*	1.4 ± 9.2	4.0 ± 7.5	4.2 ± 7.8	4.1 ± 7.5	4.1 ± 7.7

The influence of demographics on postoperative leg length differences over the course of time were investigated by a linear mixed effect model (Table 3). Significant differences of LLD measures were generally observed for the trochanter (b = − 3.15 mm (SE: 0.43) in relation to the to the tear drop figure; *P* =  < 0.001), and the iliac crest (b = 1.47 mm (SE: 0.43) in relation to the tear drop figure; *P* = 0.001). Age, BMI and gender had no significant influence on LLDs. Due to the LLD increase within week 1 and week 6 (Fig. 2), the LLD progression within the first 52 weeks can be more accurately described on a quadratic time function (b =  < − 0.01 (< 0.01); *P* < 0.001) than a linear time function (b = 0.05 mm (SE: 0.08); *P* = 0.533).

**Table 3:** Linear mixed effects on the course of leg length discrepancy (n = 99). Coefficients (b), standard errors (SE), z− values, p− values, and 95% confidence intervals given.

Measure (compared to Tear Drop Figure, in mm)	b	(SE)	z	P value	95% Conf. Interval
*Trochanter*	− 3.15	0.43	− 7.28	** < 0.001**	[− 4.00;–2.30]
*Lower Sacroiliac Joint*	− 0.42	0.43	− 0.98	0.329	[− 1.27; 0.43]
*Upper Sacroiliac Joint*	− 0.23	0.43	− 0.53	0.595	[− 1.08; 0.62]
*Iliac Crest*	1.47	0.43	3.40	**0.001**	[0.62; 2.32]
**Time (week)**	0.05	0.08	0.62	0.533	[− 0.10; 0.19]
**Age**	− 0.05	0.06	− 0.81	0.419	[− 0.18; 0.07]
**BMI**	− 0.10	0.11	− 0.08	0.937	[− 0.23; 0.22]
**Gender (male)**	− 0.55	1.07	− 0.52	0.606	[− 2.64; 1.54]
Time × time	< − 0.01	< 0.01	− 7.61	** < 0.001**	[< − 0.01; < − 0.01]
Age × time	< − 0.01	< 0.01	− 0.12	0.906	[< − 0.01; < 0.01]
BMI × time	< 0.01	< 0.01	1.50	0.134	[< − 0.01; 0.01]
Gender × time	< 0.01	0.02	0.00	0.999	[− 0.03; 0.03]
Constant	4.88	5.21	0.94	0.349	[− 5.32; 15.1]

*BMI* body mass index, *SE* standard error, *LLD* leg length discrepancy.

Furthermore, paired t-tests revealed significant differences between the trochanter in relation to the tear drop figure (difference: − 2.8 mm ± 0.7 mm; *P* < 0.001), and the iliac crest in comparison to the tear drop figure (difference: 1.8 mm ± 0.5 mm; *P* = 0.005), whilst there was no significant difference between the lower sacroiliac joint in relation to the tear drop figure (difference: − 0.4 mm ± 0.3 mm; *P* = 0.129) and the upper sacroiliac joint in relation to the tear drop figure (difference: − 0.2 mm ± 0.3 mm; *P* = 0.495).

### Change in HHS & WOMAC over time

The influence of demographic variables on HHS over time was analysed with a linear mixed model (Table 4). There was a positive influence of time from THA on HHS, both from preoperative to 12 weeks postoperative, as well as from there to 12 months postoperative (Table 4). Furthermore, male gender significantly correlated with increased HHS over time (b = 5.06 points (SE: 1.56); *P* = 0.001; Table 4). On the other hand, the use of orthopaedic arch support for perceived LLD was significantly correlated with worse HHS over time (b = − 5.72 points (SE: 1.96); *P* = 0.004). Neither LLD (b = − 0.10 points (SE: 0.39); *P* = 0.798), age (b = − 0.14 points (SE: 0.09); *P* = 0.138), BMI (b = − 0.25 points (SE: 0.17); *P* = 0.133), nor the interaction between LLD and time accomplished had a significant effect on change in HHS over time.

**Table 4:** Linear mixed effects model on change of HHS over time (n = 94). Coefficients (b), standard errors (SE), z-values, p-values, and 95% confidence intervals given.

HHS (points)		b	(SE)	z	P value	95% Conf. Interval
**Time (weeks)**	*Preop (Ref.)*					
	*12*	37.78	2.91	13.00	** < 0.001**	[32.09; 43.48]
	*52*	41.32	2.87	14.41	** < 0.001**	[35.70; 46.94]
**Age**		− 0.14	0.09	− 1.48	0.138	[− 0.32; 0.04]
**Gender (male)**		5.06	1.56	3.24	**0.001**	[1.20; 8.11]
**BMI**		− 0.25	0.17	− 1.50	0.133	[− 0.57; 0.08]
**LLD**		− 0.10	0.39	− 0.26	0.798	[− 0.88; 0.67]
**Orthopaedic arch support for perceived LLD (yes)**		− 5.72	1.96	− 2.91	**0.004**	[− 9.56; − 1.87]
Time (weeks) × LLD	*Preop (Ref.)*					
	*12*	0.43	0.43	0.99	0.321	[− 0.42; 1.28]
	*52*	0.32	0.43	0.75	0.455	[− 0.52; 1.15]
Constant		67.09	7.81	8.59	** < 0.001**	[51.78; 82.41]

*HHS* Harris Hip Score, *BMI* body mass index, *SE* standard error, *LLD* leg length discrepancy.

For the WOMAC, the linear mixed model revealed a significant decrease over time (Table 5). Similar to HHS, orthopaedic arch support used for perceived LLD showed a significant negative correlation with WOMAC over time (b = 9.77 points (SE: 3.48); *P* = 0.005). However, gender (b = − 4.04 points (SE: 2.78); *P* = 0.145), age (b = − 0.04 points (SE: 0.17); *P* = 0.815), BMI (b = 0.19 points (SE: 0.29); *P* = 0.511), LLD (b = − 0.68 points (SE: 0.59); *P* = 0.252), and the interaction between LLD and time from THA were not significantly correlated with change in WOMAC over time (Table 5).

**Table 5:** Linear mixed effects model on change of WOMAC over time (n = 94). Coefficients (b), standard errors (SE), z− values, p− values, and 95% confidence intervals given.

WOMAC (points)		b	(SE)	z	P value	95% Conf. Interval
**Time (weeks)**	*Preop (Ref.)*					
	*12*	− 34.26	4.46	− 7.69	** < 0.001**	[− 43.0; − 25.53]
	*52*	− 37.92	4.36	− 8.69	** < 0.001**	[− 46.47; − 29.37]
**Age**		− 0.04	0.17	− 0.23	0.815	[− 0.36; 0.29]
**Gender (male)**		− 4.04	2.78	− 1.46	0.145	[− 9.48; 1.40]
**BMI**		0.19	0.29	0.66	0.511	[− 0.38; 0.77]
**LLD**		− 0.68	0.59	− 1.15	0.252	[− 1.84; 0.48]
**Orthopaedic arch support for perceived LLD (yes)**		9.77	3.48	2.81	**0.005**	[2.95; 16.59]
Time (weeks) × LLD	*Preop (Ref.)*					
	*12*	0.18	0.66	0.27	0.789	[− 1.11; 1.47]
	*52*	0.35	0.65	0.53	0.593	[− 0.93; 1.62]
Constant		47.67	13.71	3.48	**0.001**	[20.80; 74.54]

*BMI* body mass index, *SE* standard error, *LLD* leg length discrepancy.

## Discussion

In the current study, an increase of leg length discrepancies over all 5 anatomical landmarks was measured during the initial postoperative phase, whereupon LLDs did not change significantly any more. The best interobserver agreements were found for the trochanter, followed by the iliac crest, and the upper sacroiliac joint. Moreover, there was an average difference of 1.8 mm and 2.8 mm in LLD as assessed on the iliac crest and the trochanter, respectively, in comparison to the tear drop figure. Therefore, it is important to report the landmark used for assessment of LLD. Harris Hip Score and WOMAC score significantly improved over time, whilst larger LLDs did not seem to negatively affect functional outcomes.

Limitations of the study include the relatively small study size of 99 patients. Therefore, currently close to but not statistically significant results may become more or less significant in case further patients could have been included. On the other hand, the results of the current study were based on over 2300 repeated measurement values taken at distinct time points, thus improving conformity. Notably, LLD measurements were exclusively performed on radiographic images rather than incorporating direct clinical methods as well, wherefore it cannot be ruled out that length differences in the femoral shaft, knee, lower leg or ankle, as well as the pelvic tilt itself, may account for LLDs observed. Although advantages and favourable precision rates of other methods to assess LLD as computed tomography, magnetic resonance imaging (MRI), or X-ray are well known^5,25–29^, all these methods may not be routinely applicable in patients following THA due to the associated high costs and radiation exposure, as well as metal artefacts in case MRI-based techniques are to be used^5,25–28^. Thus, considering the high interobserver agreement for all anatomical landmarks used, as well as the uniform and significant change of LLDs in each patient with time, the authors suppose that the chosen measurement allows reproducible and reliable objectification of LLD. Another limitation of the study is that—although a plateau in LLD measurements was observed from the 6th postoperative week onwards—it cannot be concluded with certainty from which postoperative timepoint on reliable LLD measurements may be obtained, as no additional measurements had been performed earlier than 6 weeks.

As previously described and frequently applied in clinical practice, we used plain a.p. X-rays of the pelvis to assess LLDs following THA^30–33^. Particularly the tear drop figure has been recommended in the past as a reference point to assess LLD on a.p. pelvic radiographs, as its configuration may not be significantly affected by pelvic rotation^23^. Apart from the tear drop figure, we also used the upper and lower sacroiliac joint, the trochanter, and the iliac crest to assess LLD. In line with previous findings that the lower sacroiliac joint and especially the tear drop figure may be difficult to identify following THA^34^, the best interobserver agreements were present for the trochanter, iliac crest and upper sacroiliac joint. Notably, depending on the anatomical landmark used, LLDs varied, with the largest differences seen for the trochanter to the tear drop figure, as well as the tear drop figure to the iliac crest.

Longitudinal X-ray-based measurements revealed significantly smaller LLDs at discharge in comparison to X-rays obtained at 6 weeks, whereupon LLDs did not more change significantly. Of note, due to lack of additional measurements from early postoperatively to 6th postoperative week, an exact time point upon which reliable LLD measurements may be obtained, is difficult to define. Whilst comparable changes in LLD measurements at the upper and lower sacroiliac joint as well as the tear drop figure were observed over time, those based at the trochanter and the iliac crest markedly differed. For the trochanter, which may be referred to as the reference landmark as THA does not affect leg length distal to the hip joint, stable measurements were obtained from the 6th postoperative week onwards, implying that patients did not completely extend and/or fully weight-bear the affected leg upon radiographic examination early postoperatively. At the iliac crest, on the other hand, LLD measurements on a.p. radiographs are significantly affected by pelvic torsion^35,36^, which could serve as an explanation for the marked difference in LLD measurements as compared with the lower and upper sacroiliac joint, as well as the tear drop figure.

The observation that LLD measurements on pelvic a.p. radiographs increase from discharge to the 6th postoperative week seemingly contradicts the fact that subsidence of femoral components may likewise occur during this time that would lead to a decrease in LLD^37^. However, even if subsidence occurs, it is usually small, with reported migration of 0.5 mm^38^ to 0.96 mm^39^ during the first 6 to 12 postoperative weeks. Thus, changes in patients’ postures leading to a seemingly increase in LLD during the early postoperative period may offset simultaneously occurring migration of the femoral component.

Considering that LLDs following THA are found in up to 40% of patients following THA and may even lead to a lawsuit, we further investigated whether LLD has an influence on clinical outcome scores^30,40,41^. Similar to previous studies, HHS and WOMAC improved from preoperative to the third postoperative month, and kept improving thereafter^42,43^. Notably, there was no clear correlation between change in LLD and HHS or WOMAC over time. On the other hand, male gender correlated with a significant improvement of HHS, whereas this was not the case for WOMAC. Strikingly, the use of orthopaedic arch support for patient-perceived LLD was significantly associated with poorer functional outcome, as reflected by worse HHS and higher WOMAC upon final follow-up. Similar observations have been made by *Mavcic *et al*.*^44^ and *Sykes *et al*.*^45^, with patients reporting on subjectively felt LLD presenting with worse clinical outcome scores following THA.

In conclusion, radiographic assessment of LLD following THA may not be performed early postoperatively, as measurements appear to inaccurately reflect actual LLDs at this time. However, the latest from the 6^th^ postoperative week onwards, stable and reproducible LLD measurements can be expected from plain a.p. pelvic radiographs, with the upper or lower sacroiliac joint, or tear drop figure, to be preferably used as reference landmarks.

## Data Availability

The datasets used and/or analysed during the current study are available from the corresponding author on reasonable request.

## References

[1] Gurney B (2002). Leg length discrepancy. Gait Posture.

[2] Abraham WD, Dimon JH (1992). Leg length discrepancy in total hip arthroplasty. Orthop. Clin. N. Am..

[3] Rubash HE, Parvataneni HK (2007). The pants too short, the leg too long: Leg length inequality after THA. Orthopedics.

[4] Kurtz WB (2012). In situ leg length measurement technique in hip arthroplasty. J. Arthroplasty.

[5] Sayed-Noor AS, Hugo A, Sjoden GO, Wretenberg P (2009). Leg length discrepancy in total hip arthroplasty: Comparison of two methods of measurement. Int. Orthop..

[6] Sabharwal S, Kumar A (2008). Methods for assessing leg length discrepancy. Clin. Orthop. Relat. Res..

[7] Jacobsen, S., Sonne-Holm, S. & Lund, B. Pelvic orientation and assessment of hip dysplasia in adults. *Acta Orthop Scand* **75**, 721–729 (2004). 10.1080/00016470410004094 15762262

[8] Kersic M, Dolinar D, Antolic V, Mavcic B (2014). The impact of leg length discrepancy on clinical outcome of total hip arthroplasty: Comparison of four measurement methods. J. Arthroplasty.

[9] Soderman P, Malchau H (2001). Is the Harris hip score system useful to study the outcome of total hip replacement?. Clin. Orthop. Relat. Res..

[10] Soderman P, Malchau H, Herberts P (2001). Outcome of total hip replacement: A comparison of different measurement methods. Clin. Orthop. Relat. Res..

[11] Kavanagh BF, Fitzgerald RH (1985). Clinical and roentgenographic assessment of total hip arthroplasty. A new hip score. Clin. Orthop. Relat. Res..

[12] Wright JG, Young NL (1997). A comparison of different indices of responsiveness. J. Clin. Epidemiol..

[13] Aprato A, Jayasekera N, Villar RN (2012). Does the modified Harris hip score reflect patient satisfaction after hip arthroscopy?. Am. J. Sports Med..

[14] Harris WH (1969). Traumatic arthritis of the hip after dislocation and acetabular fractures: Treatment by mold arthroplasty. An end-result study using a new method of result evaluation. J. Bone Joint Surg. Am..

[15] Riddle DL, Stratford PW, Singh JA, Strand CV (2009). Variation in outcome measures in hip and knee arthroplasty clinical trials: A proposed approach to achieving consensus. J. Rheumatol..

[16] Marchetti P (2005). Long-term results with cementless Fitek (or Fitmore) cups. J. Arthroplasty.

[17] Bellamy N, Buchanan WW, Goldsmith CH, Campbell J, Stitt LW (1988). Validation study of WOMAC: A health status instrument for measuring clinically important patient relevant outcomes to antirheumatic drug therapy in patients with osteoarthritis of the hip or knee. J. Rheumatol..

[18] Quintana JM (2005). Responsiveness and clinically important differences for the WOMAC and SF-36 after hip joint replacement. Osteoarthr. Cartil..

[19] McConnell S, Kolopack P, Davis AM (2001). The Western Ontario and McMaster Universities Osteoarthritis Index (WOMAC): A review of its utility and measurement properties. Arthritis Rheum.

[20] Stucki G (1996). Evaluation of a German version of WOMAC (Western Ontario and McMaster Universities) Arthrosis Index. Z. Rheumatol..

[21] Lumley T, Diehr P, Emerson S, Chen L (2002). The importance of the normality assumption in large public health data sets. Annu. Rev. Public Health.

[22] Portney, L. G. & Walkins, M. P. *Founations of Clinical Research: Applications to Practice* (New Jersey, 2000).

[23] Goodman, S. B., Adler, S. J., Fyhrie, D. P. & Schurman, D. J. The acetabular teardrop and its relevance to acetabular migration. *Clin. Orthop. Relat. Res. ***236**, 199–204 (1988). 3180571

[24] Castelloe, J. M. & O’Brien, R. G. *Power and Sample Size Determination for Linear Models*. 240-26 (2001). https://support.sas.com/resources/papers/proceedings/proceedings/sugi26/p240-26.pdf Accessed 2 july 2021.

[25] Aaron A, Weinstein D, Thickman D, Eilert R (1992). Comparison of orthoroentgenography and computed tomography in the measurement of limb-length discrepancy. J. Bone Joint Surg. Am..

[26] Aitken AG (1985). Leg length determination by CT digital radiography. AJR Am. J. Roentgenol..

[27] Leitzes AH (2005). Reliability and accuracy of MRI scanogram in the evaluation of limb length discrepancy. J. Pediatr. Orthop..

[28] Terry MA (2005). Measurement variance in limb length discrepancy: Clinical and radiographic assessment of interobserver and intraobserver variability. J. Pediatr. Orthop..

[29] Kjellberg M, Al-Amiry B, Englund E, Sjödén GO, Sayed-Noor AS (2012). Measurement of leg length discrepancy after total hip arthroplasty The reliability of a plain radiographic method compared to CT-scanogram. Skelet. Radiol..

[30] Konyves A, Bannister GC (2005). The importance of leg length discrepancy after total hip arthroplasty. J. Bone Joint Surg. Br..

[31] Matsuda K, Nakamura S, Matsushita T (2006). A simple method to minimize limb-length discrepancy after hip arthroplasty. Acta Orthop..

[32] Ranawat CS, Rao RR, Rodriguez JA, Bhende HS (2001). Correction of limb-length inequality during total hip arthroplasty. J. Arthroplasty.

[33] White TO, Dougall TW (2002). Arthroplasty of the hip. Leg length is not important. J. Bone Joint Surg. Br..

[34] McWilliams AB (2012). Assessing reproducibility for radiographic measurement of leg length inequality after total hip replacement. Hip. Int..

[35] Cooperstein R, Lew M (2009). The relationship between pelvic torsion and anatomical leg length inequality: A review of the literature. J. Chiropr. Med..

[36] Young RS, Andrew PD, Cummings GS (2000). Effect of simulating leg length inequality on pelvic torsion and trunk mobility. Gait Posture.

[37] Rattanaprichavej P (2019). Subsidence of hydroxyapatite-coated femoral stem in Dorr type C proximal femoral morphology. J. Arthroplasty.

[38] Kutzner KP (2020). Mid-term migration pattern of a calcar-guided short stem: A five-year EBRA-FCA-study. J. Orthop. Sci..

[39] Schaer MO (2019). Migration analysis of a metaphyseal-anchored short femoral stem in cementless THA and factors affecting the stem subsidence. BMC Musculoskelet. Disord..

[40] Edeen J, Sharkey PF, Alexander AH (1995). Clinical significance of leg-length inequality after total hip arthroplasty. Am. J. Orthop. (Belle Mead NJ).

[41] Patterson DC, Grelsamer RP, Bronson MJ, Moucha CS (2017). Lawsuits after primary and revision total hip arthroplasties: A malpractice claims analysis. J. Arthroplasty.

[42] Dong N (2016). Effect of preoperative leg length discrepancy on functional outcome and patient satisfaction after total hip arthroplasty in cases of osteonecrosis of the femoral head. J. Arthroplasty.

[43] Lindner M (2018). Psychosocial predictors for outcome after total joint arthroplasty: A prospective comparison of hip and knee arthroplasty. BMC Musculoskelet. Disord..

[44] Mavčič B, Dolinar D, Pompe B, Antolič V (2019). Patient-dependent risk factors for self-perceived leg length discrepancy after total hip arthroplasty. Eur. J. Orthop. Surg. Traumatol..

[45] Sykes A (2015). Patients' perception of leg length discrepancy post total hip arthroplasty. Hip. Int..

